# Detecting Mistakes in CPR Training with Multimodal Data and Neural Networks

**DOI:** 10.3390/s19143099

**Published:** 2019-07-13

**Authors:** Daniele Di Mitri, Jan Schneider, Marcus Specht, Hendrik Drachsler

**Affiliations:** 1 Welten Institute, Research Centre for Learning, Teaching and Technology Open University of the Netherlands, Valkenburgerweg, 177 6401 AT Heerlen, The Netherlands; 2 DIPF - Leibniz Institute for Research and Information in Education, Rostocker Straße 6, 60323 Frankfurt, Germany

**Keywords:** multimodal data, neural networks, psychomotor learning, training mistakes, medical simulation, learning analytics, signal processing, activity recognition, sensors

## Abstract

This study investigated to what extent multimodal data can be used to detect mistakes during Cardiopulmonary Resuscitation (CPR) training. We complemented the Laerdal QCPR ResusciAnne manikin with the Multimodal Tutor for CPR, a multi-sensor system consisting of a Microsoft Kinect for tracking body position and a Myo armband for collecting electromyogram information. We collected multimodal data from 11 medical students, each of them performing two sessions of two-minute chest compressions (CCs). We gathered in total 5254 CCs that were all labelled according to five performance indicators, corresponding to common CPR training mistakes. Three out of five indicators, CC rate, CC depth and CC release, were assessed automatically by the ReusciAnne manikin. The remaining two, related to arms and body position, were annotated manually by the research team. We trained five neural networks for classifying each of the five indicators. The results of the experiment show that multimodal data can provide accurate mistake detection as compared to the ResusciAnne manikin baseline. We also show that the Multimodal Tutor for CPR can detect additional CPR training mistakes such as the correct use of arms and body weight. Thus far, these mistakes were identified only by human instructors. Finally, to investigate user feedback in the future implementations of the Multimodal Tutor for CPR, we conducted a questionnaire to collect valuable feedback aspects of CPR training.

## 1. Introduction

Mastering practical skills is a requirement in several professions and working domains. For example, working in construction requires to learn how to use a circular saw; nursing requires learning how to draw blood samples from patients; and repairing clothes requires being able to sew. Practical skill training, also known as psychomotor learning, entails the acquisition of an apprenticeship learning model [1]. Typically, in this model, a human expert demonstrates to the learner how to perform a specific task. The learner mimics the expert movements to develop a mental model of the psychomotor skill and, after some practice, this model is automated. For more complex tasks, practical skills are also trained through simulation, allowing the learner to perform the task in an authentic and controlled environment. In simulations, feedback is mostly provided by the human instructor in the debriefing phase after the simulation takes place. Human instructors, however, are not always available to follow each learner step by step, and their time is costly. The lack of instructors leads to the shortage of on-task, real-time and actionable feedback affecting negatively the quality of the training and resulting in longer and less efficient training sessions for the aspiring professionals.

The shortfall of human feedback can be addressed with the employment of Intelligent Tutoring Systems (ITSs), automated computer programs designed to automatically detect learners mistakes and generate personalised adaptive feedback, without requiring the intervention of a human instructor. Although the concept of ITS dates back to decades ago (e.g., [2]), ITS interfaces have been designed as computer or web applications using the standard “mouse and keyboard" interaction paradigm, in which the learner sits in front of a computer. In contrast, practical learning activities take place primarily in physical space or, in some cases, they are complemented with the use of mobile or tablet applications. Reliable tracking of these activities, therefore, requires gathering data from multiple data sources “beyond mouse and keyboard". These various data sources can be seen as modalities of human interaction: speech, biomechanical movements, interaction with peers, manipulation of physical objects, physiological responses or contextual and environmental information. With the diffusion of smartphones, fitness trackers, wearable sensors, Internet of Things, cameras and smart objects [3], we can nowadays track human behaviour much more easily through multimodal and multi-sensor interfaces [4]. In support to the diffusion of the multimodal approach, comes as well the progress of contiguous scientific research areas such as body sensor networks [5], social signal processing [6], multimodal machine learning [7] and multimodal data fusion [8].

In the past few years, the multimodal interaction approach has become increasingly popular also in the learning science and learning analytics research communities. This increase of popularity is witnessed by the rising interest in Multimodal Learning Analytics (MMLA) [9,10]. MMLA integrates a sensor-based approach with learning analytics, to bridge learning behaviour to complex learning theories [11]. MMLA focuses on how to present learners and teachers insights from multimodal data through visualizations in analytics dashboards [12] or data-storytelling [13] in support of intuitive decision-making processes in ill-structured learning situations [14]. Recently, the MMLA community focuses more on challenges of tracking complex learning constructs with multimodal data [10]. Some of the challenges identified include data collection, storing, annotation, processing and exploitation of multimodal data for supporting learning [15].

In this study, we built upon the approach proposed by the MMLA community and further investigated how it can be applied for automatic mistake detection, which, in turn, can be the base for automatic feedback generation for *multimodal tutoring systems* in psychomotor learning scenarios. To do so, we selected cardiopulmonary resuscitation (CPR) training as representative learning task and developed an Intelligent Tutor for CPR using multimodal data (Multimodal Tutor for CPR). This study validated the mistake detection system of the Multimodal Tutor for CPR, a multi-sensor, intelligent tutoring system to train CPR using the ResusciAnne manikins.

The first objective of this study was validating the Multimodal Tutor for CPR according to common performance indicators as implemented in the ResusciAnne manikin. Thus, we used the manikin data as baseline measurements to compare the accuracy of the Multimodal Tutor for CPR to broadly established measures (*Validation*). The second objective was to explore how the Multimodal Tutor for CPR could detect mistakes that are not tracked by the ResusciAnne and are typically only detected by human instructors (*Additional mistake detection*).

CPR is a highly standardised medical procedure with clear performance indicators, therefore there are already various commercial training tools in the market which can assess most of the critical CPR performance indicators automatically. In this study, we leveraged one of these commercial tools, the ReusciAnne QCPR manikin, to derive some key performance metrics of CPR. We complemented the ResusciAnne manikin with a multi-sensor setup consisting of Microsoft Kinect to track the learner’s body position, a Myo armband for recording electromyogram data and performance indicators derived from the ResusciAnne manikin. With this setup, we ran a pilot study involving 11 experts. We used different components of the MMLA Pipeline to collect, store, annotate and process the data from the experts. We used the processed data to train five recurrent neural networks, each of them to detect common CPR training mistakes. Three of those mistakes were derived from the ResusciAnne manikin detection system while, the remaining two were annotated manually by the research team.

The paper is structured as follows. In Section 2, we introduce the CPR procedure (Section 2.1), the concept of ITSs and to what extent they were used in CPR (Section 2.2) and we explain the added value of MMLA data for CPR training (Section 2.3). Section 3 presents related studies. In Section 4, we detail the design of this study. In Section 5, we report the performance score of the classification models which we discuss in Section 6, and answer the two research questions. We also report the results of the participants’ questionnaire, aimed at collecting participants’ opinions on how to implement effective CPR feedback based on the additional mistakes detected by the models.

## 2. Background

### 2.1. Cardiopulmonary Resuscitation

Cardiopulmonary resuscitation (CPR) is a life-saving technique which is given to someone who is in cardiac arrest. CPR is useful in many emergencies, including a heart attack, near drowning or in the case of stopped heartbeat or breathing. In the present study, we selected CPR as a representative task for the following reasons:

- CPR is a procedure which can be taught singularly to one learner.
- CPR is a highly standardised procedure consisting of a series of predefined steps, that limits the set of possible actions that the learner can take.
- CPR has clear and well-defined criteria to measure the quality of the performance (we use the performance indicators defined by the European CPR Guidelines [16]).
- CPR is a highly relevant skill, which everyone should learn not only medical experts.

The cases of cardiopulmonary arrest are, unfortunately, widespread. The more people are trained to do CPR, the higher the chance of saving lives. For this reason, CPR is currently compulsory in several types of professions, and CPR training is becoming standard practice in several public settings such as schools or public workplaces.

Among the criteria for proper CPR, some indicators, such as correct CC rate, CC depth or CC depth, are more common and tracked automatically by CPR training tools such as the ResusciAnne manikins. Other CPR performance indicators are neglected to down-scale the simulation environment. For this reason, they need to be corrected by human instructors. Examples of these indicators are the use of the body weight or the locking of the arms while doing the CCs. Commercial CPR manikins such as the ResusciAnne manikin do not report on these two indicators, which creates a feedback gap for the learner and higher responsibility for the course instructors.

### 2.2. Intelligent Tutoring Systems

The intelligent tutoring system is a computer program working as “instructor in the box”, meaning that it can provide learners with direct and custom instruction or feedback, without requiring intervention from a human teacher. Traditional ITSs were mostly designed within desktop interfaces for academic education subjects such as geometry and algebra (e.g., [17,18]), and computer science subjects (e.g., [19]). In traditional academic learning, ITS has been proved to be nearly as good as human tutors [20], outperforming other instruction methods and learning activities, including traditional classroom instruction, reading printed text or electronic materials, computer-assisted instruction, laboratory or homework assignments [21]. Learning academic subjects in desktop-based interfaces is different from learning practical skills in simulations. In the former, the learner’s behaviour is represented by the words typed on the keyboard or the clicks on the correct answers. In the latter, the learner’s behaviour is determined by the interaction with physical objects using different modalities such as hands movement, gaze or speech. In practical learning scenarios, psychomotor coordination plays a much more prominent role.

In the medical field, some examples of ITS can be found in the literature. The Cardiac Tutor [22] is an ITS for training basic life support tasks such as CPR. It uses clues, verbal advice, and feedback in order to personalise and optimise the learning process. The Collaborative Medical Tutor (COMET) [23] an intelligent tutoring system for medical problem-based learning that focuses on learners collaboration.

Only a few examples of ITS use multimodal interfaces. D’Mello et al. [24] enhanced the ITS AutoTutor with a multisensor interface consisting of eye-tracking and posture sensor embedded in the chair where the learner is sitting, so it can detect learners’ affective and cognitive states. A comparable setup was also used by Burleson [25] in the Affective Learning Companion, using a camera for face recognition, learner posture, wrist-based skin conductivity and mouse pressure to detect learners’ affective states during game playing. In a more recent study, Schneider et al. [26] used a Kinect-based system in the Presentation Trainer, an ITS for training public presentation skills which give both real-time as well as retrospective feedback about the quality of the presentation. Another example is the Calligraphy Tutor by Limbu et al. [27], which uses EMG and capacitive pens for training calligraphy and writing in a foreign language.

### 2.3. Multimodal Data for Learning

In the context of education and learning, with the term *multimodal data*, we refer to learner’s motoric movements, physiological responses, information of the learning context, environment and activity. Combining data from multiple modalities allows obtaining a more accurate representation of the learning process [28]. Multimodal data, therefore, can be used as historical evidence for the analysis and the description of the learning process [9]. Multimodal data can be collected using wearable sensors, cameras, Internet of Things (IoT) devices, and computer logs. Research in the field shows there are several existing devices which can be used in the field of learning for collecting data and prompting feedback [29].

In the field of MMLA, related studies have used multimodal data to investigate learning performances. In the context of classroom activities, Raca and Dillenbourg [30] analysed student attention from posture and computer vision. D’mello et al. [31] tracked teacher–student dialogues interaction using audio data. Domínguez et al. [32] used a tracking device, the *Multimodal Selfie*, to analyse video audio pen strokes of each student.

In group collaboration settings, Ochoa et al. [33] collected multimodal data including video, audio and pen strokes, to classify expertise. In [11], researchers recorded video and audio from 13 students building simple structures with sticks and tape. From video data, they derived skeletal position and gesture movements, translating the multimodal transcripts into *action codes* and task-specific patterns.

In the MMLA literature, it is possible to identify two approaches to MMLA: (1) the “analytics” approach which aims at presenting insights to the educational actors, helping them to provide a better feedback, particularly useful in ill-structured learning tasks [14]; and (2) the “modelling” approach, used also by the ITS community, which aims at using machine learning to automatically classify or predict learning dimensions. There are, however, examples that combine the two approaches (e.g., [26,34]), which suggest there is not a “black and white” division but rather a continuum between the ITS and MMLA fields.

In the latter, the intelligent algorithms are fed with the multimodal data associated with task performance measurements and are trained to classify or predict learning goals, training mistakes and prompt on-time automatic and personalised feedback. The modelling approach in the field of MMLA, is described by a recent model, the *Multimodal Learning Analytics Model* (MLeAM) [35].

Although the potentials of MMLA for learning are well documented, practical applications still remain a challenge. Multimodal data are messy. To get meaningful and supportive interpretations from multimodal data, intensive data geology steps are required. Moreover, to the best of our knowledge, no standardised procedures exist in this field. In this study, we use an emerging approach, the Multimodal Learning Analytics Pipeline (MMLA Pipeline) [36], in the context of CPR training for creating Multimodal Tutor for CPR. We believe that the selected method can be used as road map for the general identification of learners mistakes using multimodal data in authentic training procedures.

## 3. Related Studies

As CPR is a life-saving technique, there is a prosperous research community around the topic, which also publishes in CPR-specific resuscitation journals and conferences. Scouting the related literature, we also found that the idea of using Kinect-based systems for tracking CPR is not new. The study by Semeraro et al. [37] first piloted a Kinect-based system for providing feedback on CC depth noticing that the depth camera is well suited for the CPR task and that Kinect-based system can improve performance. In the study of Wattanasoontorn et al. [38], the authors analytically programmed an algorithm to detect the arm posture and CC rate, a weak part of the approach was the calibration process needed to make the detection work. The study in [39] designed a Kinect-based real-time audiovisual feedback device to investigate the relationship among rescuer posture, body weight and CC quality. They tested 100 participants monitoring depth and rate of CC and providing further real-time feedback. The result of this study is that kneeling posture provides better CC than a standing posture and that audio-visual feedback can provide a better CC depth, rate, and effective CC ratio. In our study, we proposed the use of a neural network to detect training mistakes in terms of CC rate, CC depth, and CC release, as well as to detect additional training mistakes, not currently tracked by commercial manikins, such as the correct locking of the arms during and the correct use of body posture and body weight during CC. Moreover, in the setup we proposed, we also included a Myo armband to prove the concept of a system learning from multiple modalities. Differently from the previous studies that use tailor-made solutions, the Multimodal Tutor for CPR is a multimodal system that uses generic solutions for data collection described in the MMLA Pipeline.

## 4. Method

This study validated the mistake detection system of the Multimodal Tutor for CPR, a multi-sensor, intelligent tutoring system to train CPR using the ResusciAnne manikins. First, we aimed at validating the Multimodal Tutor for CPR on performance indicators currently implemented in the ResusciAnne using the manikin data as baseline measurements of the CPR performance (RQ1—*Validation*). After that, we explored if the Multimodal Tutor for CPR could detect mistakes not tracked by the ResusciAnne but typically only detected by human instructors (RQ2—*Additional mistake detection*). Our research questions therefore are:

RQ1 *Validation*: How accurately can we detect common mistakes in compression rate, compression depth and release depth in CPR training with multimodal sensor data in comparison to the ReusciAnne manikin?
RQ2 *Additional mistake detection*: Can we use multimodal data to detect additional CPR training mistakes such as “locking of the arms” and use of “body weight” which are not tracked by ResusciAnne manikin and are only identified by human instructors?

To answer these research questions, we conducted a quantitative observational study in collaboration with AIXTRA, simulation centre of the Uniklink in Aachen, Germany. The experiment involved collecting data from 11 participants. We focused on the quality criteria of the CCs, which is a part of the procedure of the CPR. Each participant performed two sessions of two minutes continuously doing CC, without rescue breaths. For answering RQ1, we used the ResusciAnne manikin data as the baseline measurement to validate the correct mistake detection of the Multimodal Tutor for CPR. To answer RQ2 and mark the presence of additional mistakes in the CPR executions, the research team annotated manually the recorded sessions.

### 4.1. Experimental Setup

**Figure 1:**
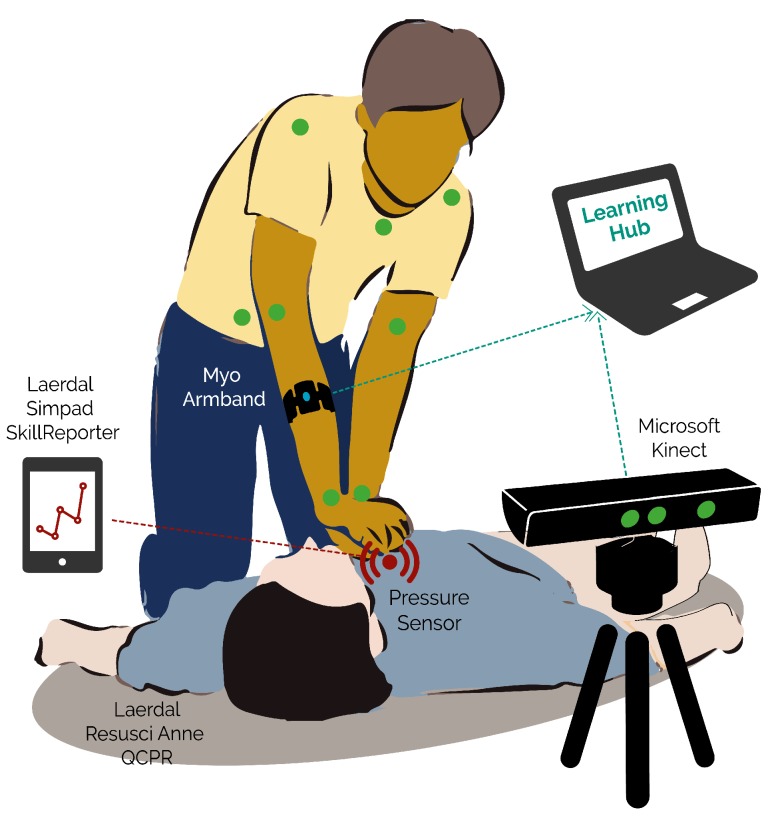
The graphic representation of the experimental setting.

The multimodal setup, as represented in Figure 1, consisted of the following devices:

- A Microsoft Kinect (v2), depth camera capable of recording three-dimensional skeleton position of the expert, and video record the expert.
- A Myo armband, a Bluetooth device which records electro-myogram and accelerometer data of the person wearing it and provides haptic feedback.
- A Laerdal ResusciAnne QCPR full body manikin, a popular CPR training manikin optimised for multiple feedback.
- A Laerdal Simpad SkillsReporter, a touchscreen device that couples wirelessly with the ResusciAnne manikin and allows to debrief the overall performance CPR through the assessment of multiple CPR indicators.

The ResusciAnne manikin and its SimPad SkillsReporter are validated CPR instruments, which allow extracting high-quality performance data and they are guideline-compliant according to the official ECR guidelines [16]. We used the indicators derived from the SimPad device as our baseline for measuring the quality of the CPR training performance and answer RQ1. On the SimPad device, we used the two-minute-long CC in “evaluation mode”.

Among the data that can be retrieved from the SimPad SkillsReporter, we considered the following indicators (the first three in Table 1): (1) CC rate; (2) CC depth; and (3) CC release. The remaining two indicators ((4) Arms position; and (5) body position) are not tracked by the SimPad SkillsReporter.

### 4.2. Participants

CPR is a standard training procedure which requires to be taught by certified trainers. For this reason, we decided that Multimodal Tutor for CPR, at least in the prototyping phase, was not suitable to test complete beginner but rather participants who had previous training knowledge. We selected 14 experts, advanced medical and medical dentist students of the Uniklink Aachen University Hospital. As evidenced in the questionnaire (reported in Section 5.3), each participant followed, on average, five CPR training courses. All participants were asked to sign an informed consent letter, describing the experimental details, as well as the data protection guidelines, elaborated following the new European general data protection regulation (GDPR 2016/679). The data collected from the participants were fully anonymised.

### 4.3. Experimental Procedure

The experimental procedure consisted of three phases: (1) prototyping phase; (2) on-site experiment; and (3) analysis. In the first phase, before the on-site experiment, we designed and improved the Multimodal Tutor for CPR iteratively. One or multiple data collection sessions involving one participant, and consequent analysis of the quality of the data collected followed each design iteration improvement of the system. When the prototype reached a satisfactory level, we organised an on-site experiment in cooperation with the University Hospital. Participants were tested individually. Each test consisted of two sessions of two minutes doing CCs separated by a five-minute break, during which the participant had to answer a questionnaire. We recorded each two-minute session separately. Phase 3 was the in-depth data analysis of the data collected. In this phase, we discarded the data from 3 out of 14 participants (6 out of 28 sessions) due to insufficient quality—either caused by faulty Myo readings or incorrect booting of the LearningHub. We narrowed the number of participants considered in the data analysis to 11, for a total of 22 sessions. To identify mistakes “arms properly locked” and correct “body weight”, we also recorded five extra sessions with mistakes conducted on purpose by one of the participants.

The technological approach used for the experiment is the MMLA Pipeline [36] a workflow for the collection, storage, annotation, analysis and exploitation of multimodal data for supporting learning. We used three existing component implementations of the MMLA Pipeline. In the next sections, we describe how we used each of these existing components in the experiment of the Multimodal Tutor for CPR.

### 4.4. Data Collection

The data of each session were recorded using the Multimodal Learning Hub [40] (LearningHub), a system for data collection and data storing of multimodal learning experiences using multiple sensors applications. It focuses on short and meaningful learning activities (∼10 min) using a distributed, client–server architecture with a master node controlling and receiving updates from multiple sensor data provider applications. Each sensor application retrieves data updates from a single device, it stores it into a list of frames, and, at the end of the recordings, it sends the list of frames to the LearningHub. In this way, the LearningHub allows collecting data from multiple sensors streams produced at different frequencies. Already various sensor applications have been implemented to work with the LearningHub, both for commercial devices as well as for custom sensor boards. The LearningHub and its sensor data provider applications have all been implemented Open Source [41]. In the Multimodal Tutor for CPR, the LearningHub was used together with three sensor applications, the Kinect data provider, the Myo data provider and a screen recorder. The Kinect data provider collected data from 15 body joints represented as three-dimensional points in space and position features for a total of 60 attributes. The Myo data provider collected accelerometer, orientation, gyroscope and readings from eight EMG sensors for a total of 18 attributes. The screen recorder captured the video of the participant performing the CCs through the point of view of the Kinect. For the data collection, we also used the ResusciAnne manikin data recorded with the SimPad, which provided the baseline assessment of the CPR performance of the participants. This device was not integrated with LearningHub but saved on the local memory of the SimPad, hence transferred via USB to the computer used for the analysis. The SimPad sessions were then manually synchronised with the help of the Visual Inspection Tool (see Section 4.6) [36]. The most sensitive data, the video recording of the participants, were only included during the annotation phase, exclusively by the research team. The video recordings were eventually taken out from the sessions files making the dataset entirely anonymous. During the experiment, we asked each participant to fill in a short questionnaire soon after the first session. The questionnaire aimed at collecting additional information about the participant’s level of previous expertise. We asked questions regarding previous training, kind of feedback received, pros and cons of such feedback and self-perceived performance during the first CPR session. The purpose of this questionnaire was also to gain extra information on how to build useful feedback for the Multimodal Tutor for CPR. The results of this questionnaire are detailed in Section 5.3.

### 4.5. Data Storage

The LearningHub uses the concept of *Meaningful Learning Task* introducing a new data format (MLT session file) for data storing and exchange. The MLT session comes as a compressed folder including: (1) one or multiple time-synchronised sensor recordings; amd (2) one video/audio of the recorded performance. The sensor recordings were serialised into JSON and have the following properties: an applicationId, an applicationName and a list of frames. The frames have a timestamp and a key-value dictionary of sensor attributes and their corresponding values. In the Multimodal Tutor for CPR, each two-minute CPR session was recorded into a separate MLT session file and stored locally. Each session was 17 Mb and contained initially two JSON files, one for the Myo, one for the Kinect and one MP4 file with the video recording. The example of Myo and Kinect is presented in table view in Table 2 and Table 3.

### 4.6. Data Annotation

The CPR annotations were later synchronised with the sessions using the Visual Inspection Tool (VIT) [36]. VIT allows the manual and semi-automatic annotation of psychomotor learning tasks which can be captured with a set of sensors. The VIT enables the researcher: (1) to triangulate multimodal data with video recordings; (2) to segment the multimodal data into time intervals and to add annotations to the time intervals; and (3) to download the annotated dataset and use the annotations as labels for machine learning classification or prediction. In addition, the VIT is a software released under Open Source license [42]. The annotations created with the VIT are saved into MLT data-format as the other sensor files. The annotations were treated as an additional sensor application, where each frame is a time interval with relative startTime and stopTime instead of a single timestamp. Using the standard MLT data-format, the user of the VIT can both define custom annotation schemes or load existing annotation files.

**Figure 2:**
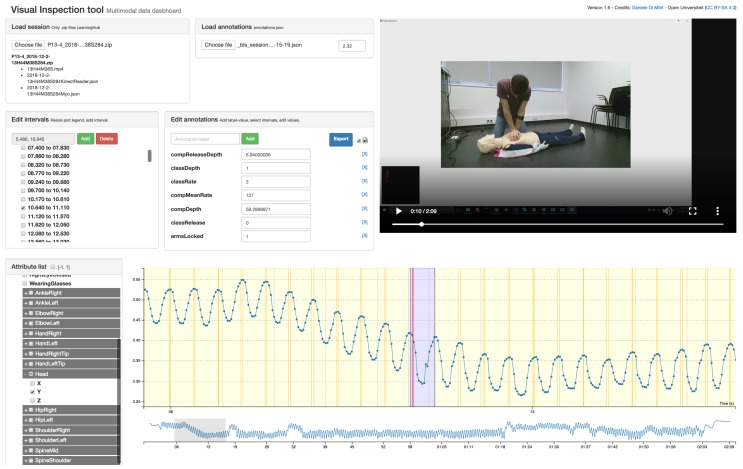
Screenshot of the VIT.

**Figure 3:**
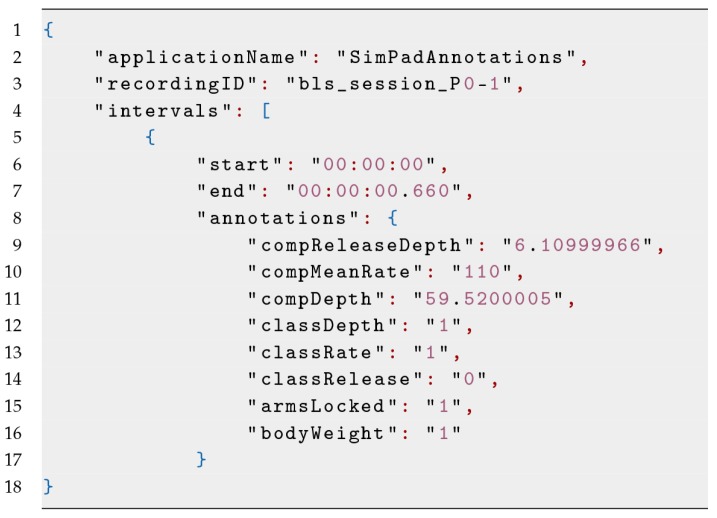
Example of the annotation file derived from the SimPad and loaded in the VIT.

A screenshot of the VIT used for the Multimodal Tutor for CPR is given in Figure 2. We derived the annotation files by the sessions of the SimPad converted into MLT data format. An example of such file is shown in Figure 3 and in Table 4, having an annotation attribute of the three indicators discussed in Table 1. As we added the annotation file manually using the VIT, the time-intervals were not synchronised with the other sensor recording. The VIT, however, allowed setting a time offset to align the annotations manually to the sensor recordings. For each of the 22 CPR sessions recorded, we loaded the corresponding annotation file, and synchronised it manually, being guided by the sensor data plots and the video recordings. Hence, we downloaded the annotated session, excluding the video file.

The classification scheme used for the performance indicators summarised in Table 1 was based on their ideal interval and on the feedback that the learner would receive. In the case of CC rate, the value of the CC can be either lower than the interval (Class 0) or within the interval (Class 1) or above the interval (Class 2). If labelled with Class 0, the CC rate is “too slow”, and then the feedback should be to increase the speed of the CC. If Class 2, the CC rate is “too fast”, and then the feedback should be to decrease the speed of the CC. With Class 1, the CC rate is “on point”. A similar approach is for CC depth: Class 0 the depth is “too shallow,” and the feedback should be to “push harder”; Class 2 is too deep, and the feedback should be to “push less hard”; and Class 1 indicates the CC depth is “on point”. CC release, arms position and body position follow instead a binary classification approach, either the task is correctly executed (Class 1) or not correctly executed (Class 0). Once again, we based this decision on the type of feedback the learner should receive, which can be “release the CC completely”, “lock your arms” or “use your body weight correctly”.

### 4.7. Data Analysis

The 22 annotated datasets were loaded into a Python script using Numpy and Pandas, two data manipulation libraries widely used for statistical analysis. These libraries allow the user to define custom data frames with custom time-indexes ideal for time-series and perform various kinds of vectorised operations. The Python script implemented the following routine. It created a list of all the available sessions, and then iterated and processed each session singularly. The results of the processing are stacked up into a single data frame.

#### 4.7.1. Preparing the Data

The script processes first the annotation file, in order to have all the intervals (i.e., the CCs) into a single data frame $DF_{intv}$. In the MLT format, the annotation file is different from other sensor files as it is the only JSON file having a list of intervals with start and end, instead of frames with timestamps. The script also computes the duration of the interval subtracting *end* with *start*. As the session implement relative timing, where $t_{0}=0$, it is important to add the session date-time to the timer to differentiate between sessions. Consequently, the script processes each of the sensor JSON files. It transforms the list of frames each one with the same set of attributes into a table having the frames as rows and the attributes as columns. It removes the underscores and other special characters from the attribute names and it adds the time-offset. It sets the timestamp as the index and removes the duplicates with the same index. It discards all the attributes whose running total equals zero, which are uninformative attributes. Note that this approach is only applicable to numerical and not categorical data. Finally, it concatenates the results into one single, time-ordered data frame $DF_{attr}$. From such data frame, some attributes are excluded a priori, as considered of not being informative. In our case, we excluded the data from ankles, hips and head. The hips and ankles as the participants are on their knees when doing CPR and heads because sometimes people tend to raise their heads to look up, right or left, which is a movement not influencing the quality of the CCs. At the end of the iteration, we have two data frames:

- $DF_{intv}$, in which the rows $\left(t_{0}..t_{n}\right)$ are time intervals (i.e., CCs) and $\left(y_{0},\ldots ,y_{m}\right)$ columns are the target indicator values (annotations).
- $DF_{attr}$, in which the rows $\left(j_{0},\ldots ,j_{p}\right)$ are sensor updates and $\left(a_{0},\ldots ,a_{q}\right)$ columns are the sensor attributes. As the sensor applications have different update frequencies, $DF_{attr}$ is a sparse matrix having many zeros.

We proceeded to mask $DF_{attr}$ with the time intervals $\left(t_{0}..t_{n}\right)$, in order to create a new data frame:

- $DF_{mask}$, which has *n* elements, $\left(x_{0}..x_{n}\right)$. Each element is an array of $\left(a_{0},\ldots ,a_{q}\right)$ time series of about 0.5 s containing the sensor updates for that specific time interval.

**Figure 4:**
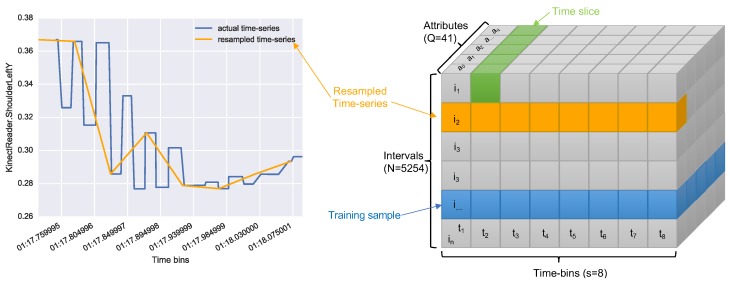
(**Left**) Example resampling of a time-series interval of the attribute *Kinect.ShoulderLeftY*. (**Right**) A graphic representation of the data transformation into a tensor of shape (5254×8×41).

The issue with this was that time series in $DF_{mask}$ were of different sizes, not smoothed and with missing values. For this reason, we resampled each of them with equal size (S=8). An example of this resampling is shown in Figure 4 (left). The resampling process led us to a tensor of size (N×S×Q) where *N* is the number of intervals, *S* is the size chosen for the resampling, and *Q* is the number of attributes. Figure 4 (right) shows a graphical representation of the tensor obtained.

#### 4.7.2. Training the Neural Networks

For machine learning, we opted for using Keras, an open source neural network library written in Python. We used the Keras implementation of the *Long Short Term Memory networks (LSTM)* [43], a type of recurrent neural network (RNN) able to learn over long sequences of data. RNNs are looping networks in which each iteration leaves a footprint, which is used to calculate the following iterations. For this reason, RNNs retain a memory of the previous iterations. When the network updates, however, the memory degrades with a vanishing gradient which can result in the loss of valuable information. To address this issue, LSTM networks use additional long-term memory, where important information are stored to prevent them from degrading over time. The advantage of using LSTM in the CPR domain is able to preserve relevant information throughout the entire CPR session.

We first scaled the tensor’s values in a range between 0 and 1. Then, the following sub-routine was applied three times for each target class *classRate*, *classDepth*, and *classRelease*. Then, we split the dataset in 66.6% training and 33.4% test using random shuffling of the training samples. Then, the data were fed into the LSTM neural network with two layers:

1. LSTM input layer of size (8×41) feeding into consisting of a hidden layer of 128 units; and
2. dense layer sized as unique values of the target class (either 2 or 3).

**Figure 5:**
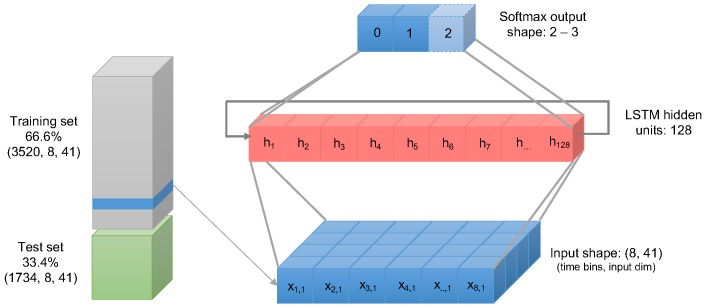
Graphical representation of the LSTM neural network configuration.

In Figure 5, we present a graphical representation of the configuration of the LSTM neural network. We compiled the LSTM neural network with the training dataset selecting 30 fitting iterations (epochs). As model parameters, the loss function was set using the Sparse Categorical Cross-Entropy was chosen, while we set the accuracy as the performance metrics to evaluate the model. The model was also evaluated using the remaining 33.4% of the dataset. The results obtained are discussed in Section 5.

## 5. Results

The dataset transformed into a tensor of shape (5254×8×41) where 5254 are the learning samples, 41 the attributes and 8 the fixed number of time updates (time-bins) for each attribute. As the ratio chosen between split and test was 66/33, the training set consisted of 3520 samples while the test set in 1734 samples.

The annotation data retrieved from the ResusciAnne manikin are summarised in Table 5. The data refer to the total across the 11 participants. While the mean value for compression depth 54.49 mm and for compression release 4.74 mm matches with the CPR guidelines in Table 1, the mean compression rate was 121.59 compression/minute, which is slightly above the guideline’s range. For this reason, the training mistake which participants seemed to make most often was to compress the chest too fast.

**Figure 6:**
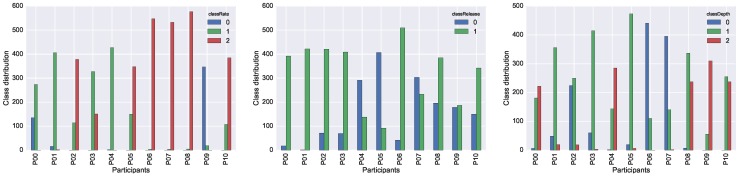
Class distribution for each individual participant for: *classRate* (**left**); *classRelease* (**centre**); and *classRelease* (**right**).

We report the individual performance of each participant in the three plots in Figure 6. In the case of *classRate* and *classDepth*, the target variables with three possible class-values, it is interesting to acknowledge that each participant shifted almost always in two classes out of three. Participants tended to make only one type of mistake for a particular target, or, in other words, either they were too fast or too slow, but not a combination of both. The performance of a single participant is therefore not representative for the full span of possible mistakes that may occur during training. A more even distribution of mistakes can only be achieved when collecting data from multiple participants.

### 5.1. Neural Network Results

**Figure 7:**
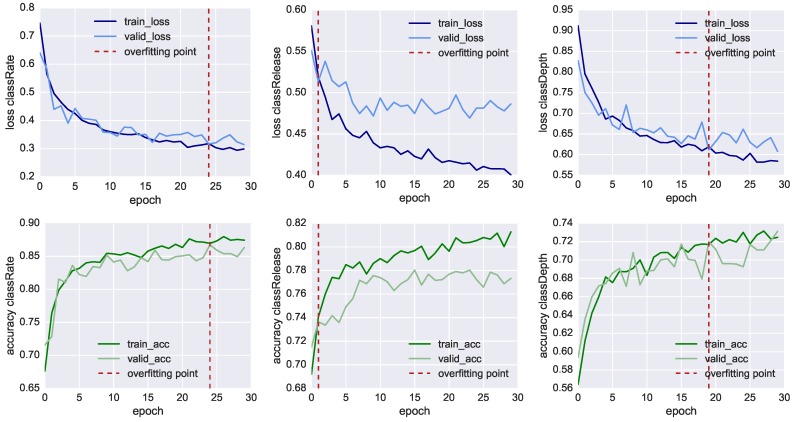
Performance values of the three classifiers. Loss function (**top**) and Accuracy (**bottom**) of the classifiers: *classRate* (**left**); *classRelease* (**centre**); and *classRelease* (**right**), during training (dark line) and validation (light line). Dashed red lines indicate the “overfitting points”, i.e., the epoch in which training loss continues decreasing while the validation loss does not improve anymore.

In Figure 7, we plot the results of the neural network training of the three classifiers, showing both the loss function values (charts in the top row) and the model’s accuracy (charts in the bottom row) through 30 fitting iterations (also called epochs). At each iteration, we compare the results of the training set using 3520 samples (dark line) with the result of the validation set using 1734 samples (light line). We also mark with the red dashed line the inflection point which highlights when the model starts overfitting the training data. In this point, the training loss function (dark blue line) reduces progressively, whereas the validation loss (light blue line) remains more or less stable. In this way, it is possible to identify the iteration where the training of the model has to be stopped before it starts overfitting the data. For *classRate*, this epoch is around the 24th iteration, for *classDepth* around the 19th, and for *classRelease* around the 2nd.

Nonetheless, in Table 6, we can see all the three classifier reached an accuracy higher than 70% before overfitting. The most accurate model is the one classifying *classRate*, the binary class indicating if the CCs were executed at the right rate and speed, followed by *classDepth*, indicating the correct depth of the compression, and *classRelease*, indicating the correct release of the CC. To achieve better accuracy, lower loss function and less premature overfitting, we would need to have more training data.

With an accuracy of 86.5%, *classRate* is the best-classified target. A plausible explanation is that the depth-camera sensors, as well as the accelerometer embedded in the Myo, can track temporal-related features such as the acceleration in doing the compression. It is essential to point out that all three models shared the same set of 41 features, all CCs were re-sampled into eight bins and we did not take the actual duration of each CC as additional feature. The reason *classRelease* and *classDepth* do not perform as well as *classRate* could stem from the fact that the compression and the release are movements of few centimetres and the threshold of correctness is thin to measure, even for a human observer.

**Figure 8:**
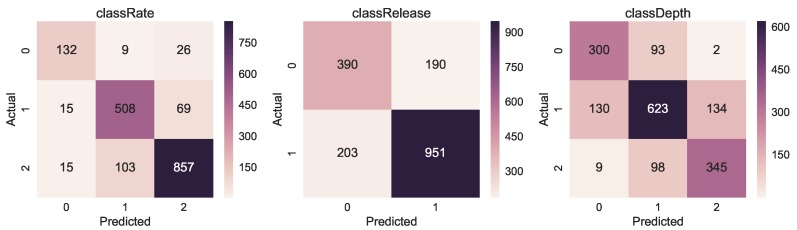
Confusion matrices for *classRate*, *classDepth* and *classRelease*. The *y*-axis is the actual class, while the x-axis is the predicted class.

**Figure 9:**
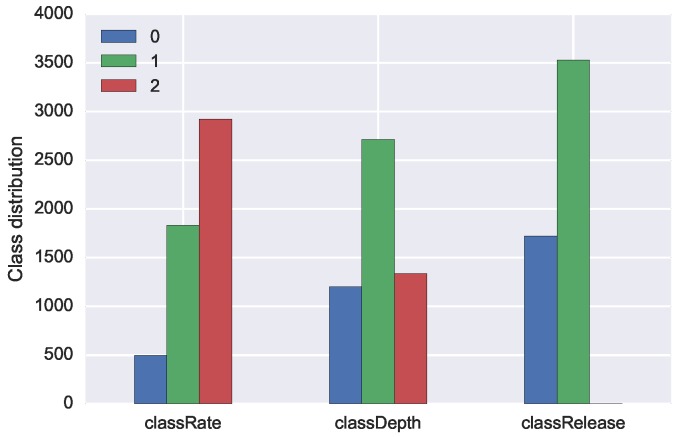
Overall class distribution for *classRate*, *classDepth* and *classRelease*.

The confusion matrices in Figure 8 present the result of correct and incorrect classifications of the three models. The tendency we can observe is that the highest number of correct classification (darker colour) happens for class with the highest number of examples (see Figure 9 for reference).

### 5.2. Manually Annotated Classes

For the two additional CPR training mistakes of arms not correctly locked (*armsLocked*) and failing to use entire body weight (*bodyWeight*), we used an additional dataset consisting of five sessions with one participant mimicking the two mistakes. The reason we opted for this solution was because all participants in the initial data collection showed very good CPR technique and did not commit these two types of errors.

**Figure 10:**
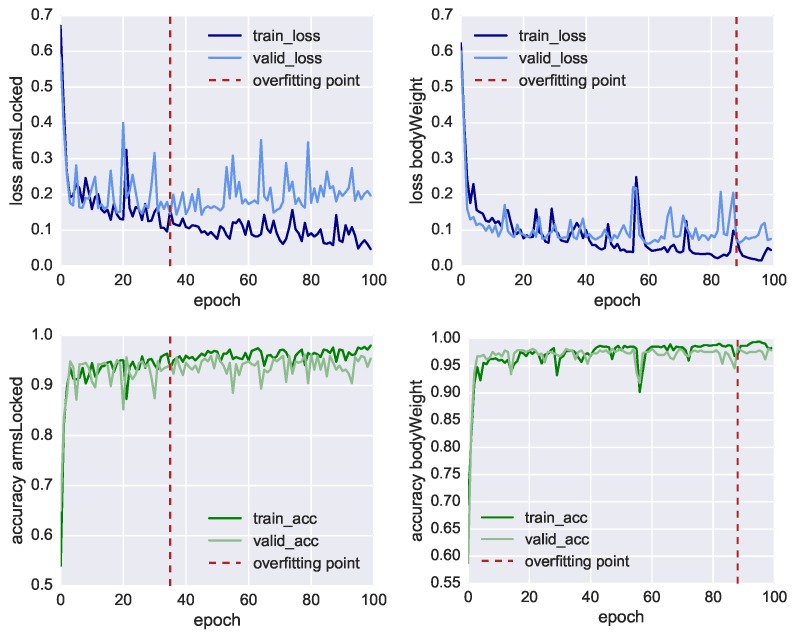
Performance values of the classifiers for the two manually annotated classes *armsLocked* and *bodyWeight*. Loss function (**top**) and Accuracy (**bottom**) of the classifiers: *armsLocked* (**left**); and *bodyWeight* (**right**), during training (dark line) and validation (light line). Dashed red lines indicate the “overfitting points”, i.e., the epoch in which training loss continues decreasing while the validation loss does not improve anymore.

**Figure 11:**
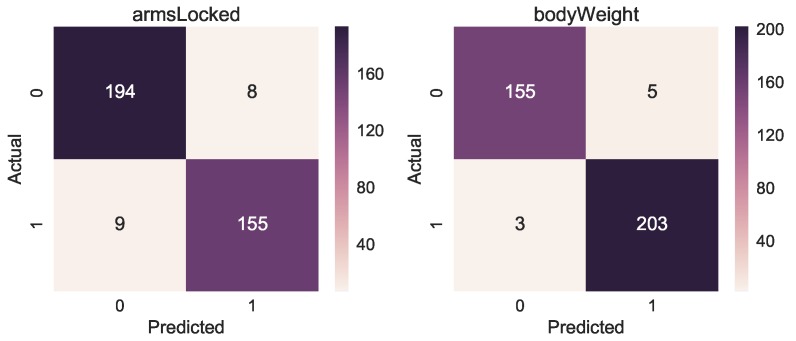
Confusion matrices for the manually annotated classes *armsLocked* and *bodyWeight*.

For the new dataset, we used the same methodology described in Section 4. We collected in this case 1107 CCs, 41 attributes and 8 time bins leading to a tensor of size (1107 × 8 × 41). We trained two LSTM neural networks for 100 iterations, the performances are shown in Figure 10 both for training and validation loss and accuracy showing also the overfitting point. Before the model starts overfitting, we achieved for *armsLocked* 93.4% accuracy and for *bodyWeight* 97.8% accuracy, as shown in Table 7. We also show for both target classes the Area Under the Receiver Operating Characteristic Curve (ROC AUC), obtaining 93.7% and 98.3%, respectively. The accuracy of the manual annotation is higher due to the fact that the training errors of arms not correctly locked and incorrect body weight are easier to track with a depth camera. The confusion matrices in Figure 11 also show the results of the correct or incorrect classifications.

### 5.3. Questionnaire Results

**Figure 12:**
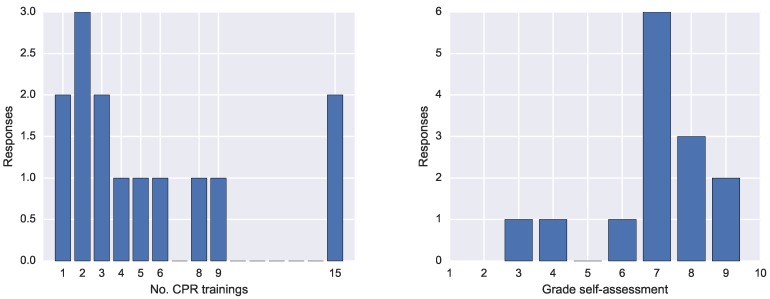
(**left**) Answers on for Question 1, the number of previous CPR trainings for each participant; and (**right**) the self-assessment ratings given by the participants to their performance.

The questionnaire entailed seven questions and had 14 respondents. The first question concerned the number of CPR trainings. The answers spanned from 1 to 15 trainings, as shown o in Figure 12 (left).

The second question required an open answer and it concerned the type of feedback received during those training. All respondents answered they had received verbal feedback from the CPR trainer. Three respondents mentioned having received feedback also directly from the manikin, and two from the device connected to the manikin reporting the performance. One respondent mentioned the feedback was also written other than verbal, another that was also visual. Three mentioned the feedback was given in real-time feedback while two others retrospectively.

The third question asked about what was the most crucial aspect of the feedback received. Nine respondent agreed that the feedback from the instructor was the most important and that was because the expert also makes an imitation of either the training mistake or the correct position. Another mentioned aspect was to be able to revise their own performances looking at the and depth and frequency of the CCs.

The fourth question asked why the feedback was useful. The answers were more diverse, including to get a better understanding of how to execute CPR optimally; helping to keep calm during emergency; the instructor showing the corrections to adopt; the possibility to correct mistakes on time; and being reminded to not lose strength. A respondent answered that it is difficult to realise their own mistakes while another asserted that “even if you know the rules, practising in reality is different”.

The fifth question asked about any missing aspect of the feedback received in the previous CPR trainings. Three respondents mentioned lack of information of the depth compression, while other respondents remarked the need for real-time feedback because the CPR procedure is tiring and during execution there is little awareness of the perceived performance.

The sixth question asked the participants desired feedback in CPR. The most mentioned was real-time audio (five times), then real-time visualisations, dashboards at the end and haptic vibrations or augmented reality visuals.

Finally, we asked the participants to rate their CPR performance with a grade from 1 to 10. The results in Figure 12 show that the most frequent grade was 7, followed by 8 and 9.

## 6. Discussion

With the results presented in Section 5, we can answer the two research questions of the study as outlined in Section 4.

RQ1 focused on the validation of the Multimodal Tutor for CPR and the collected multimodal data against the standardised ResusciAnne manikin measurements (RQ1—*Validation*). With the approach described in Section 4, we were able to train three models based on recurrent neural networks, capable of classifying the right compression rate with 86% accuracy, correct compression release with 73% accuracy and correct compression depth with 71% accuracy. Therefore, we can answer RQ1 positively, the Multimodal Tutor for CPR and the collected multimodal data can detect standardised mistakes in CPR training almost as accurately as common CPR training manikins. We also need to point out that the models were not heavily optimised, which makes us confident that they can be improved to achieve even higher accuracy scores.

Concerning the generalisability across learners, all models trained to classify training mistakes tracked with the ResusciAnne manikin generalised well across different users. Training for each individual participant would also be possible, however, in that case, the data points would have been only around 400 instead of 5200. That would have resulted in higher accuracy score, but also a higher risk of over-fitting the training data. In general, as CPR is a standard procedure that requires the same and repetitive movements, the individual differences do not seem to hold a strong influence for the classifiers. This condition, however, applies only when the experimental setup is unchanged. In the case the setup changes, then generalising becomes more difficult. For example, if the Kinect were moved to a different location, the values of the sensors would be quite different, or, if a sensor were either added or taken out from the current setup, it would result in a set of different features. A limitation of the approach used therefore is the need to keep the learning activity setup as unchanged as possible.

RQ2 focused on the classification of training mistakes that so far can only be detected by the human instructors and not by the ResusciAnne manikin (RQ2—*Extended mistake detection*). We specifically focused on locked arms and body position mistakes to be detected by the Multimodal Tutor for CPR. To answer this question, we trained our model on additional CPR session where the two training mistakes, *bodyWeight* and *armsLocked*, were mimicked. We achieved an accuracy score of 93% for *armsLocked* and 97% for *bodyWeight*. Based on this high accuracy, we can answer RQ2 positively: the Multimodal Tutor for CPR was able to detect additional CPR training mistakes beyond the mistake detection of the standard ResusciAnne manikin. As with the previous research question, a limitation of our approach is that the set-up must remain unchanged.

## 7. Conclusions

In this paper, we introduce a new approach for detecting CPR training mistakes with multimodal data using neural networks.

The CPR use case was chosen as representative learning task for this study being a standardised and relevant skill. In fact, improving CPR learning feedback could reduce training time and make the CPR procedure more efficient, which would result in greater support for people having a cardiopulmonary arrest and consequently a higher number of lives saved. We designed the Multimodal Tutor for CPR, a multi-sensor setup for CPR, which is a specific implementation of the Multimodal Tutoring System, consisting of a Kinect camera and a Myo armband. We used the Multimodal Tutor for CPR in combination with the ResusciAnne manikin for collecting data from 11 experts doing CPR. We first validated the collected multimodal data upon three performance indicators provided by the ResusciAnne manikin, observing that we can classify accurately the training mistakes on these three standardised indicators. We can further conclude that it is possible to extend the standardised mistake detection to additional training mistakes on performance indicators such as correct locking of the arms and correct body position. Thus far, these mistakes could only be detected by human instructors. After these positive findings regarding the abilities of the Multimodal Tutor for CPR, we envision a follow-up study to investigate different feedback interventions for the learners during CPR training. To facilitate this further research, we asked the participants to fill-in a questionnaire to elicit the most relevant aspects of the feedback they received during CPR training the elements which they considered most useful. These principles can be used for the future study of the Multimodal Tutor for CPR, along with the models trained to detect mistakes. In addition, a run-time feedback engine which ensures the multimodal data are captured in real-time and that the feedback is timely.

With the *Multimodal Tutor for CPR*, we demonstrate that multimodal data can be used as the base to drive machine reasoning and adaptation during learning. This can be used both for automatic feedback (modelling approach) as well as for retrospective human feedback (analytics approach).

Among the findings to our research questions, we can also report that the *MMLA Pipeline* for collecting multimodal data from various sensors (see Section 4.1) and our MMLA approach for automatically identifying CPR training mistakes, has proven to be highly effective. We are convinced that the suggested approach can be extended to other psychomotor learning tasks. To prove this claim in future works, Multimodal Tutoring Systems for additional psychomotor learning domains need to be developed. 

## Figures and Tables

**Table 1:** Considered CPR performance indicators and whether they are detected by the SimPad SkillsReporter and by the human instructor.

Performance Indicator	Ideal Value	SimPad	Instructor
CC rate	100 to 120 compr./min	✓	✓
CC depth	5 to 6 cm	✓	?
CC release	0–1 cm	✓	?
Arms position	elbows locked	✗	✓
Body position	using body weight	✗	✓

**Table 2:** Table view of the Myo armband data; (2) Kinect camera data; and (3) data from Annotation.json, table view of the JSON in shown in Figure 3.

frameStamp	Myo.EMGPod0	...	Myo.EMGPod7	Myo.GyrX	Myo.AccZ	Myo.OriY
00:00:02.0160129	−5.0	...	−4.0	53.0625	−0.137695	−0.199036
00:00:02.0170122	−4.0	...	0.0	45.5625	−0.343750	−0.186706
00:00:02.0297305	−2.0	...	−4.0	45.5625	−0.343750	−0.186706

**Table 3:** Table view of the Kinect camera data.

frameStamp	Kinect.ElbowLeftX	Kinect.ElbowRightY	Kinect.HandLeftX	Kinect.HeadZ	...
00:02.0282288	2.076761	0.275064	−0.520056	1.995384	...
00:02.0607683	2.057799	0.258791	−0.513448	1.996636	...
00:02.0932877	2.019825	0.249333	−0.502858	1.998549	...

**Table 4:** Table view of the JSON in shown in Figure 3.

start	end	classDepth	classRate	classRelease	compDepth	compMeanRate	compRelease
00:07.070	00:07.730	1	1	0	59.520001	110	6.11
00:07.750	00:08.380	2	0	1	60.910000	98	4.00
00:08.390	00:08.770	1	0	1	60.000000	97	2.00

**Table 5:** Annotation data from the SimPad summarised across participants.

Indicator	Mean	Std	Min	Max
compDepth (mm)	54.49	6.00	60.22	62.94
compMeanRate (compression/min)	121.59	16.08	133.0	164.0
compReleaseDepth (mm)	4.74	3.64	6.11	30.0
duration (sec)	0.44	0.06	0.48	0.88

**Table 6:** Accuracy scores, loss values and ROC-AUC scores for each of the target classes.

	Test accuracy	Test loss	ROC-AUC score
*classRate*	0.8650	0.3241	n.a.
*classRelease*	0.7391	0.5121	0.7305
*classDepth*	0.7180	0.6144	n.a.

**Table 7:** Accuracy scores, loss function values and ROC-AUC score for the two manually annotated classes.

	Test Accuracy	Test Loss	ROC-AUC Score
*armsLocked*	0.9344	0.1595	0.9371
*bodyWeight*	0.9781	0.0694	0.9833

## Abbreviations

The following abbreviations are used in this manuscript:

CPR	Cardiopulmonary Resuscitation
CC	Chest compression
EMG	Electromyogram
GDPR	General Data Protection Regulation
JSON	JavaScript Object Notation
RQ	Research Question
MLT	Meaningful Learning Task
VIT	Visual Inspection Tool
MMLA	Multimodal Learning Analytics
ITS	Intelligent Tutoring System
MLeAM	Multimodal Learning Analytics Model
